# Gradual adoption of needle biopsy for breast lesions in a rural state

**DOI:** 10.1002/cam4.4282

**Published:** 2021-11-09

**Authors:** Serena Murphy, Yi‐Chuan Yu, Colleen Kerrigan, Brian Sprague, Michelle Sowden

**Affiliations:** ^1^ Department of Surgery University of Vermont Medical Center Burlington VT USA; ^2^ College of Agricultural and Life Sciences University of Vermont Burlington VT USA; ^3^ Department of Surgery Vermont Breast Cancer Surveillance System University of Vermont Burlington VT USA

**Keywords:** breast cancer, needle biopsy, population, quality improvement, standard of care

## Abstract

**Background:**

Minimally invasive breast biopsy (MIBB) is the standard of care for the diagnosis of breast cancer, with consensus guidelines suggesting MIBB goals of 90% of total biopsies. In a previous study of patients in the rural state of Vermont, USA (population size of 640,000), rural breast cancer patients had open biopsies 42% of the time compared to 29% of urban breast cancer patients. The aim of this study was to assess overall population‐based biopsy trends in Vermont.

**Methods:**

The Vermont Breast Cancer Surveillance System (VBCSS) was used to identify women receiving MIBB and excisional breast biopsies in Vermont. Patient zip code at the time of initial biopsy was used to determine the patient residence rurality by rural–urban commuting area codes (RUCA 2.0™).

**Results:**

There were 9122 diagnostic episodes from 1999 to 2018. MIBB was the initial biopsy method in 7524 (82.5%) cases, while surgical excision was the initial biopsy method in 1598 (17.5%) cases. A linear trend fit estimated an increase of 1.3% per year (*p* < 0.001, 95% CI 1.1%–1.5%) in the fraction of patients undergoing MIBB. Patients living in rural areas were less likely to receive MIBB (78.5%) than those living in urban areas (94.9%), *p* < 0.001. Multivariate analysis showed that urban patients and those patients in the years 2014–2018 were more likely to receive MIBB (OR 5.00, 95% CI 4.13–6.05 [*p* < 0.05] and OR 4.41, 95%CI 3.68–5.28 [*p* < 0.05], respectively). The rate of MIBB for rural patients increased and met the 90% quality standard in 2013 and ultimately matched urban patient rates of MIBB in 2018.

**Conclusions:**

For the first time, we show that MIBB usage is above 90% in the state of Vermont and that there no longer exist disparities in breast biopsies between urban and rural patients or rural/urban facilities in the state, overall.

## Graphical abstract

Minimally invasive needle biopsy has been the standard of care for breast cancer diagnosis since the early 2000’s. However, a number of studies through 2010, including our prior report from Vermont, showed persistently high rates of open surgical biopsies in rural populations. In this manuscript, we report updated results from a state‐wide retrospective study of breast biopsies, stratified by rurality of patient residence; we evaluated the trend of minimally invasive breast biopsies from 1999 to 2018 and show for the first time that the state as a whole has reached the standard of care of over 90% breast biopsies performed as minimally invasive.

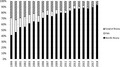

## 1 INTRODUCTION

Minimally invasive breast biopsy (MIBB), which includes core needle and fine‐needle aspiration (FNA), has been considered the standard of care for the diagnosis of breast cancer since the early 2000s in the United States. Several studies have confirmed the accuracy of MIBB in diagnosing both benign and malignant breast lesions.^1^, ^2^, ^3^ Minimally invasive biopsies confer significant advantages when compared to open excisional biopsies. Patients that undergo MIBB suffer less morbidity as a result of their procedure, are less likely to undergo unnecessary surgery, are more likely to have negative margins at the time of their first operation, and are more likely to undergo only one surgery for their cancer.^4^, ^5^ There is substantial cost saving to both the patient and the health network with MIBB since open excisional biopsy requires an expensive, time‐consuming procedure in an operating room.^6^, ^7^ While there are special circumstances in which open excisional biopsies are permissible, the clear benefits of MIBB have led several consensus guidelines since 2001 to suggest rates of MIBB to meet or exceed 90% and that open excisional biopsy should be the rare exception.^8^, ^9^ Similarly other parts of the world have issued guidelines regarding transitioning standard of care to minimally invasive biopsies; in 2010 the European Society of Breast Cancer Specialists (EUSOMA) suggested that 90% of all women with breast cancer should have a preoperative diagnosis by means of percutaneous biopsy.^10^

As breast cancer care becomes increasingly complex, MIBB is even more important to be considered among breast cancer patients. The diagnosis of cancer preoperatively allows evaluation of prognostic factors including estrogen and progesterone receptor status of the tumor, Her2 status, grade, and type of cancer.^11^ This allows physicians to tailor therapeutic drugs based on the biology of cancer. Preoperative tissue diagnosis also allows for additional staging work‐up and genetic testing, if needed. This information can then be discussed in a multidisciplinary fashion with medical and radiation oncologists prior to surgical intervention. There are several subsets of breast cancer where neoadjuvant chemotherapy (chemotherapy given prior to surgery) has become much more common and in some cases is the standard of care. An open biopsy for diagnosis eliminates the option for neoadjuvant or intraoperative therapies and the usefulness of multidisciplinary preoperative discussion.^12^, ^13^, ^14^

Unfortunately, several studies show that patients in rural communities are receiving open biopsies at much higher rates than their urban counterparts. Medicare claims data showed that from the years 2001 to 2008 patients in rural Texas were receiving open biopsy up to 43% of the time compared to 26% among their urban counterparts.^15^ Similarly, analysis of national trends in the Healthcare Cost and Utilization Project National Inpatient Sample Database from 2008 to 2010 showed that rural patients were significantly more likely to have open biopsy than their urban counterparts (36% vs. 29%).^16^ This same conclusion was found in a statewide analysis of women in the rural state of Vermont during 1998–2006, which showed that rural patients had an open biopsy 42% of the time compared with 29% of urban MIBB patients.^5^ These findings raised significant concerns about overall high rates of surgical biopsy as well as rural disparities in access to breast biopsy standard of care.

Since the previous study of Vermont‐patient breast biopsy data reported from James et al in 2012 showed MIBB rates well below the 90% goal for the study years of 1998–2006,^5^ there have been no studies to indicate if there has been improvement in the state. It is unclear whether patterns in breast biopsy have changed in the past 10 years since these prior studies. The aim of this study is to look at the most recent trends in surgical and MIBB in the rural state of Vermont. We examined overall trends in the state over time, patterns by patient urban/rural residence, and facility‐level trends at thirteen healthcare facilities in the state. In this study, we anticipate that the overall population‐based biopsy trends would have MIBB rates of 90% or greater since 2006. Our goal is to assess the types of breast biopsies in our state, which will further inform our best practices among breast cancer patients for the state of Vermont and similar rural states in the US.

## 2 METHODS

### 2.1 Study design

Approval for this study was given by the University of Vermont Institutional Review Board (IRB). To identify women receiving minimally invasive and excisional breast biopsies in the state of Vermont, we utilized the Vermont Breast Cancer Surveillance System (VBCSS). The VBCSS is a registry collecting data from the thirteen radiology and eight pathology laboratory facilities in Vermont that perform breast imaging and evaluate breast specimens, respectively. The VBCSS collects patient and radiology data for all patients undergoing breast imaging at radiology facilities in Vermont and pathology reports for all breast specimens evaluated at pathology facilities in Vermont. Pathology reports are collected by the VBCSS for all breast specimens evaluated at pathology facilities in Vermont (not a sample). All pathology reports are abstracted into the VBCSS database, including information on the type of biopsy. Direct patient identifiers are included with radiology and pathology data. Patient matching algorithms are used to link data across facilities, such that women who undergo procedures at one facility are linked to their breast care records from another facility. The patient, radiology, and pathology data from these facilities are linked to consolidated breast cancer records from the Vermont Cancer Registry. The participating institutions include one academic tertiary care medical center (the University of Vermont Medical Center), seven critical access hospitals (CAHs), and five community hospitals (CHs).

A trained abstractor enters data from breast pathology reports into the VBCSS database, including type of biopsy. Diagnostic episodes were defined to begin with breast biopsy (FNA, core needle, or surgical excision) that was not preceded by a separate breast biopsy within the same breast in the past year. The first biopsy procedure in each diagnostic episode was recorded as the biopsy type and classified as surgical excision or MIBB (FNA or core needle). After internal validation, procedures performed by plastic surgeons were excluded to ensure exclusion of excisional procedures performed for cosmetic purposes. If a woman had more than one diagnostic episode, each diagnostic episode was tabulated as a separate record in the analytic dataset. Analysis was limited to diagnostic episodes between 1999 and 2018 that resulted in a breast cancer diagnosis. Women with a prior history of cancer and women with breast implants were excluded from analyses.

Patients were characterized based on a standardized health questionnaire administered at the breast imaging exam preceding the biopsy. The questionnaire had an “opt‐out” option. Only 8% of the participants in this study were opt‐out. Counts and proportions of biopsies by type (surgical excision vs. MIBB) were grouped by the year of the procedure was done and the facility at which the biopsy procedure was performed. The patients’ zip code at the time of the initial biopsy was used to determine the rurality of the patient's residence. This was accomplished by using rural–urban commuting area codes (RUCA 2.0™), which classify U.S. census tracts using measures of urbanization, population density, and daily commuting. Individual zip codes of patient residence and the location of the health care facility at which the initial breast biopsies were performed were assigned RUCA codes 1–10 in the dataset, as per published resources.^17^ The most urban is labeled as “1” and the most rural is labeled as “10.” RUCA codes 1–3 were classified as urban while 4–10 were classified as rural. This urban‐rural classification is based on the rural‐urban classification system for community health assessment from the Washington State Department of Health, USA.^17^

### 2.2 Statistical analysis

Statistical analysis was carried out using StataIC 15™. A chi‐square test was calculated to determine if the patient demographics were equally distributed between the biopsy techniques. All *p* values reported are two‐sided. A *p*‐value of < 0.05 was determined to be significant and 95% confidence intervals were determined for proportions using the binomial exact method. A linear regression model was used to determine the rate of MIBB increase across the years studied. Finally, we used logistic regression to evaluate the likelihood of undergoing MIBB according to urban versus rural residence. The models were adjusted for patient‐level demographics and characteristics including previous breast biopsy, age, education, and year of biopsy.

## 3 RESULTS

An initial sample size of 10,558 diagnostic episodes were identified between 1999 and 2018. After exclusion criteria were applied (procedures performed by plastic surgeons, women with a previous history of breast cancer, and women with breast implants), 9122 were available for analysis. MIBB was the initial biopsy method in 7524 (82.5%) cases, while surgical excision was the initial biopsy method in the remaining 1598 (17.5%) cases. Of the total 7524 MIBB procedures, 88.4% (*n* = 6649) were needle biopsies and 11.6% (*n* = 875) were FNAs.

Demographics show that 96.1% of the study population was non‐Hispanic white, while 1.3% was Hispanic. The remainder were non‐Hispanic minorities (Table 1). Across all years in the study, the youngest patients (<40) had the highest rates of MIBB (84.8%) while older patients (80+) had the lowest rates (76.1%). Those without a high school degree were most likely to have a surgical biopsy (25.3%). As the years progressed in the study, diagnostic episodes were more likely to be MIBB; from 2014 to 2018 the diagnostic episodes were 91.5% MIBB. Overall, patients undergoing surgical biopsy were more likely to be older and have less education.

**TABLE 1:** Characteristics of women with breast cancer who underwent breast biopsy in Vermont, 1999–2018

	All women *N* = 9122		MIBB *N* = 7524	% MIBB	Surgical *N* = 1598	% Surgical	*p* Value
	*n*	%	*n*		*n*		
Age							<0.001
<40	363	4.0	308	84.8	55	15.2	
40–49	1671	18.3	1397	83.6	274	16.4	
50–59	2310	25.3	1920	83.1	390	16.9	
60–69	2249	24.7	1902	84.6	347	15.4	
70–79	1683	18.5	1353	80.4	330	19.6	
80+	846	9.3	644	76.1	202	23.9	
Year of biopsy							<0.001
1999–2003	2328	25.5	1673	71.9	655	28.1	
2004–2008	2308	25.3	1857	80.5	451	19.5	
2009–2013	2299	25.2	1993	86.7	306	15.3	
2014–2018	2187	24.0	2001	91.5	186	8.5	
History of breast biopsy							0.002
No	6029	78.3	5088	84.4	941	15.6	
Yes, same breast	1016	13.2	813	80.0	203	20.0	
Yes, opposite breast only	655	8.5	550	84.0	105	26.0	
Unknown	1422	(15.6)	1073	75.5	349	24.5	
Education							<0.001
<High school diploma	643	7.7	480	74.7	163	25.3	
High school diploma	2355	28.3	1917	81.4	438	18.6	
Some college	1873	22.5	1584	84.6	289	15.4	
College degree	3457	41.5	3003	86.9	454	13.1	
Unknown	794	(8.7)	540	68.0	254	32.0	
Urban/rural residence							<0.001
Urban	2548	31.7	2417	94.9	131	5.1	
Rural	5490	68.3	4312	78.5	1178	21.5	
Unknown	1084	(11.9)	795	73.3	289	26.7	

**FIGURE 1:**
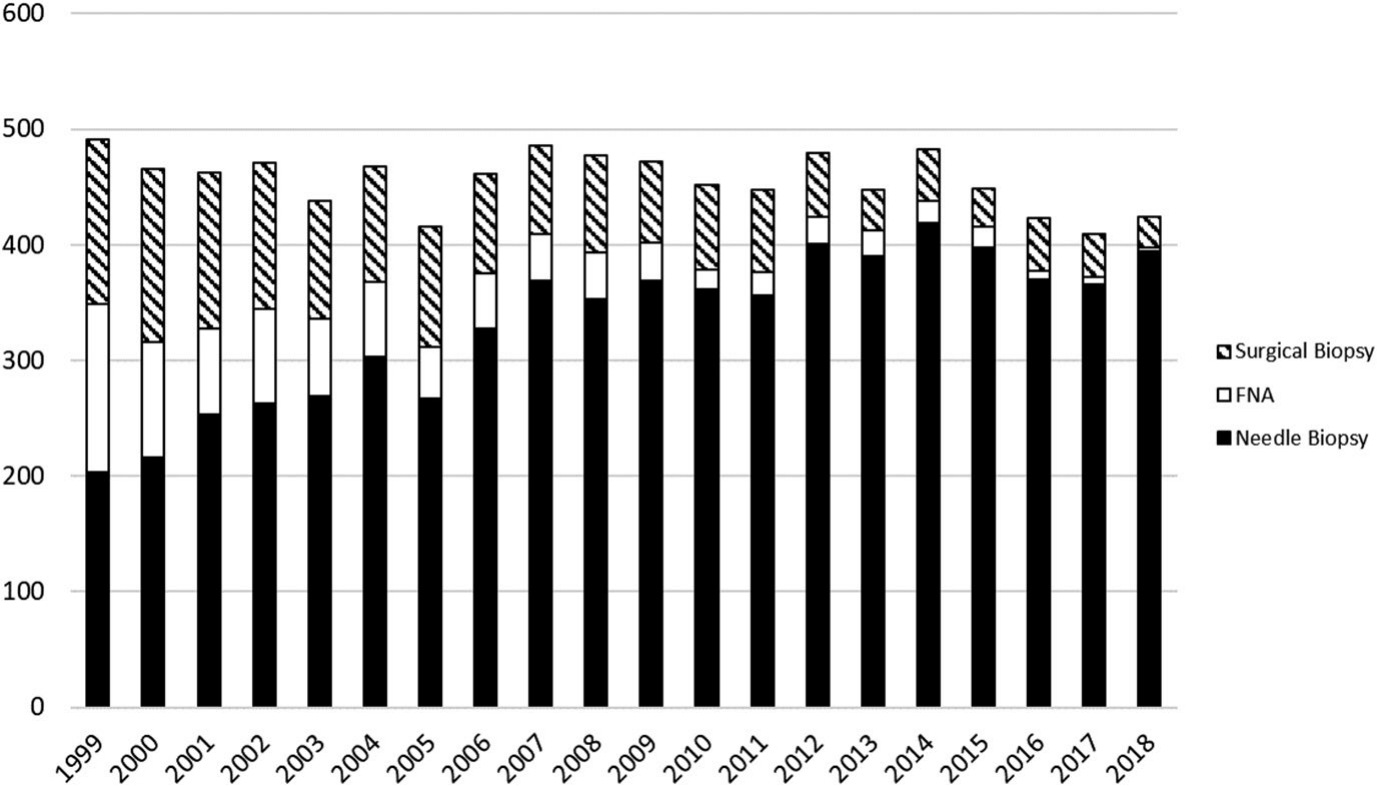
Biopsy type by year in Vermont

Figure 1 and Table S1 show the biopsy type performed in Vermont by year. There was an increase in the use of MIBB over the study period time, with the fraction of patients undergoing MIBB increasing steadily between 1999 and 2018 (71.9% in 1999–2003, 80.5% in 2004–2008, 86.7% in 2009–2013, and 91.5% in 2014–2018; Table 1). A linear trend fit to the data estimated an average absolute increase of 1.3% per year (*p* < 0.001, 95% CI 1.1%–1.5%) in the fraction of patients undergoing MIBB. While surgical excision constituted nearly one‐third of the biopsies performed in Vermont in the year 1999, by the year 2013, the surgical biopsy rate fell below 10%.

**FIGURE 2:**
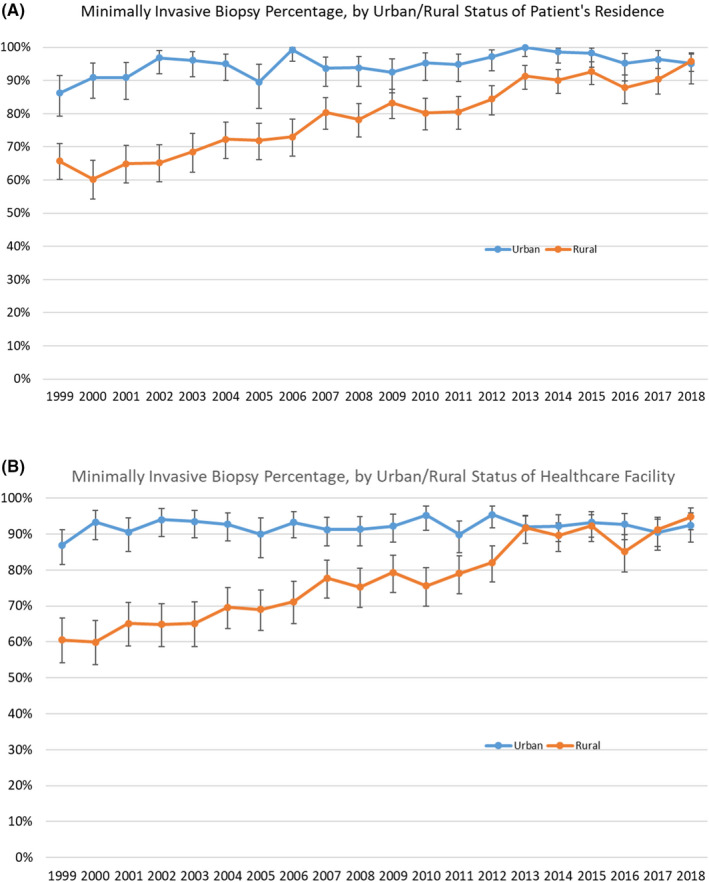
(A) Percent of biopsies that were minimally invasive, by year and urban/rural status of patient's residence. Error bars depict 95% confidence interval. (B) Percent of biopsies that were minimally invasive, by year and urban/rural status of facility location. Error bars depict 95% confidence interval

Patients living in rural areas were less likely to receive a MIBB (78.5%) than those living in urban areas (94.9%) (Table 1; *p* (chi‐squared) <0.001). Multivariate analysis showed the OR of urban women receiving MIBB was 5.00, 95% CI 4.13–6.05 [*p* < 0.05] (Table 2). However, the MIBB proportion steadily increased over the study period in both the rural and urban patient categories (Figure 2A, Table S2A), with an OR of 4.41, 95%CI 3.68–5.28 [*p* < 0.05] for women receiving MIBB in the years 2014–2018. As time progressed, the rate of MIBB in the rural patient category increased and met the 90% quality standard in 2013 and ultimately matched the urban patient rate of MIBB in 2018. When characterized by urban or rural facility location (Figure 2B, Table S2B), the trends remained the same, with rural facilities reaching above 90% MIBB by 2013. Though, as seen in the trend for patients in rural residences, the rural facility MIBB rate does drop briefly in 2016 below the national standard before rising again in 2017.

**TABLE 2:** Multivariable‐adjusted logistic regression estimates of odds ratios for minimally invasive breast biopsy

Factor	Odds ratio (95% confidence interval)
Age	
<40	1.30 (0.94–1.80)
40–49	1.03 (0.86–1.23)
50–59	Ref
60–69	1.06 (0.89–1.25)
70–79	0.88 (0.74–1.05)
80+	0.77 (0.63–0.95)
Year of biopsy	
1999–2003	Ref
2004–2008	1.64 (1.42–1.89)
2009–2013	2.57 (2.20–3.01)
2014–2018	4.41 (3.68–5.28)
History of breast biopsy	
No	Ref
Yes, same breast	0.76 (0.63–0.91)
Yes, opposite breast only	0.97 (0.77–1.22)
Education	
<High school diploma	0.65 (0.52–0.81)
High school diploma	0.88 (0.75–1.02)
Some college	0.98 (0.83–1.15)
College degree	Ref
Urban/rural residence	
Urban	5.00 (4.13–6.05)
Rural	Ref

**FIGURE 3:**
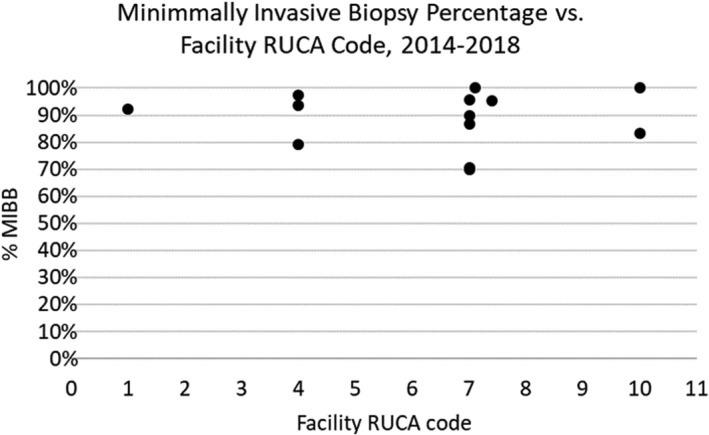
Percent of initial biopsies that were minimally invasive, by year and facility, where specimen was obtained

The MIBB percentage over time is shown for individual facilities in Table S3. There were five facilities that were found to be below the national standard of 90% minimally invasive biopsy in the most recent 2014–2018 interval, each of which had a RUCA code of 4 or greater (Figure 3). Three of those facilities were found to have less than 80% of their biopsies to be MIBB.

## 4 DISCUSSION

This study inspected trends in MIBB and surgical biopsy rates in Vermont. We observed a pattern toward increasing rates of MIBB with corresponding decreasing rates of excisional biopsies from 1999 to 2018. By 2006, over 80% of total breast biopsies in the state of Vermont were performed minimally invasively, and by 2013, the national standard of at least 90% of breast biopsies being performed minimally invasively was met by Vermont, overall. However, when broken down by location, five facilities were still found to be behind the national standard; all five of these facilities were found to have rural designations per RUCA guidelines. Interestingly, three of those facilities that were below 80% were noted to be radiology facilities that do not have stereotactic biopsy capabilities. The reasons behind the slower uptake of needle biopsy cannot be completely explained since this study did not address this but is thought to be multifactorial. But there have been similar trends in other geographical areas. One study based in Ontario, Canada surveyed 385 general surgeons and found that surgeons opted for excisional biopsy after consideration of factors such as delayed access to MIBB, patient preference for open excision, and low clinical suspicion for malignancy. Other factors, such as lack of equipment or expertise, were found less likely to influence a surgeon's decision.^18^ A separate multivariate analysis of 3644 women in Ontario showed that the local health network and surgeon/radiologist specialization were predictive of women receiving MIBB.^19^ A 2015 study of Medicare claims data in Texas showed that there were both facility and provider impacts on MIBB rates, namely smaller, low volume hospitals had higher rates of excisional biopsy as did older, foreign‐trained surgeons with lower case volumes.^20^ What cannot be obtained from this study are the nuanced discussions between surgeons and patients regarding required follow up and patient preference.

When stratifying the rurality of the patients who received breast biopsies in Vermont between 1999 and 2018, we found that those patients living in rural areas have nearly 4‐fold higher rates of surgical excisional biopsies than those patients living in urban areas. Our rural patients have an open surgical biopsy technique in 21.5% of cases versus the 5.1% of urban patients across all years of this study, which reflects previously published disparities in rural and urban surgical biopsy rates.^15^, ^21^, ^22^ However, there is finally a trend toward MIBB in our study, showing MIBB to be above 90% for both urban and rural patients, an improvement since the last investigation in our state.^5^ And when broken down by year, in the years 2013–2018, there ceases to be a rural/urban disparity in MIBB rates in all years except 2016 (Figure 2A). Overall, there have been improvements in the MIBB rates to reach national guideline recommendations of 90% in both the urban and rural populations of this state; this speaks to the changes that have been made in the rural facilities since the last publication.^5^ These results will help guide new policies within our health network which will help provide direct feedback to facilities that have not met the 90% MIBB rate.

Our study has few limitations. First, VBCSS is a statewide registry, which receives data only on procedures that are performed within the state of Vermont. Women who received biopsies in Vermont but have surgical procedures outside of the state do not have complete data within the database. Additionally, the patient characteristics are self‐reported from patient information forms; the collection of this data is based on an “opt‐out” system. Up to the most recent report, only 8% of patients are opt out. This study also cannot determine the details surrounding the clinical diagnosis, especially concerning the reason behind surgical excisional biopsies from patients or physicians. Justification to surgical excisional biopsies include lesions not amenable to image‐guided biopsy or patients unable to maintain the positioning required for minimally invasive biopsies. There is a similar justification for high‐risk pathologies, such as atypical ductal hyperplasia. Our inclusion of only breast cancer diagnoses intended to account for these scenarios for excisional biopsy.

## 5 CONCLUSION

To improve patient satisfaction, reduce unnecessary procedures, and decrease health care costs, the American College of Surgeons recognizes MIBB as the standard of care for initial diagnostic biopsy for suspicious breast lesions.^8^, ^9^ For the first time, our study shows that the most recent MIBB usage is above 90% in the state of Vermont and the disparities in breast biopsies between urban and rural patients or rural/urban facilities is no longer a concern. While there are five facilities that still fall below 90%, overall rural facilities have shifted away from surgical breast biopsies and rates below the national standard may be due to specific facility resources. Increased attention is needed for both providers and hospitals toward the placement of resources toward specific facilities for needle biopsy capabilities.

## CONFLICT OF INTEREST

No conflict of interest disclosures from any authors.

## ETHICAL APPROVAL STATEMENT

The protocol and procedures employed were ethically reviewed and approved by the UVM IRB.

## Supporting information

Table S1–S3

## Data Availability

The data that support the findings of this study are available from the corresponding author upon reasonable request.
