# Supplementary material for: Gradual adoption of needle biopsy for breast lesions in a rural state
Source: Cancer Med. 2021 Nov 9;10(23):8320–7. doi: 10.1002/cam4.4282 (PMC8633243; doi:10.1002/cam4.4282)
Supplement: Supplementary file 1 — Table S1–S3 [file CAM4-10-8320-s001.docx]

| **Supplemental Table 1: Types of Breast Cancer Biopsy by Year, Vermont Breast Cancer Surveillance System** | | | | | | | |
| --- | --- | --- | --- | --- | --- | --- | --- |
| Year | Surgical Biopsy | | FNA | | Needle Biopsy | | Total |
|  | N | (%) | N | (%) | N | (%) |  |
| 1999 | 142 | 28.9 | 146 | 29.7 | 203 | 41.3 | 491 |
| 2000 | 150 | 32.2 | 100 | 21.5 | 216 | 46.4 | 466 |
| 2001 | 134 | 29.0 | 75 | 16.2 | 253 | 54.8 | 462 |
| 2002 | 127 | 27.0 | 81 | 17.2 | 263 | 55.8 | 471 |
| 2003 | 102 | 23.3 | 67 | 15.3 | 269 | 61.4 | 438 |
| 2004 | 100 | 21.4 | 65 | 13.9 | 303 | 64.7 | 468 |
| 2005 | 104 | 25.0 | 45 | 10.8 | 267 | 64.2 | 416 |
| 2006 | 86 | 18.7 | 47 | 10.2 | 328 | 71.1 | 461 |
| 2007 | 77 | 15.8 | 40 | 8.2 | 369 | 75.9 | 486 |
| 2008 | 84 | 17.6 | 40 | 8.4 | 353 | 74.0 | 477 |
| 2009 | 70 | 14.8 | 33 | 7.0 | 369 | 78.2 | 472 |
| 2010 | 73 | 16.2 | 17 | 3.8 | 362 | 80.1 | 452 |
| 2011 | 72 | 16.1 | 20 | 4.5 | 356 | 79.5 | 448 |
| 2012 | 55 | 11.5 | 23 | 4.8 | 401 | 83.7 | 479 |
| 2013 | 36 | 8.0 | 22 | 4.9 | 390 | 87.1 | 448 |
| 2014 | 44 | 9.1 | 19 | 3.9 | 419 | 86.9 | 482 |
| 2015 | 33 | 7.3 | 18 | 4.0 | 398 | 88.6 | 449 |
| 2016 | 46 | 10.9 | 7 | 1.7 | 370 | 87.5 | 423 |
| 2017 | 37 | 9.0 | 6 | 1.5 | 366 | 89.5 | 409 |
| 2018 | 26 | 6.1 | 4 | 0.9 | 394 | 92.9 | 424 |
| Total | 1,598 | 17.5 | 875 | 9.6 | 6,649 | 72.9 | 9,122 |

| **Supplemental Table 2A: MIBB Percentage According to Urban/Rural Status of Patient's Residence** | | |
| --- | --- | --- |
| Year | Urban (%) | Rural (%) |
| 1999 | 86.2 | 65.7 |
| 2000 | 90.9 | 60.2 |
| 2001 | 90.9 | 64.9 |
| 2002 | 96.8 | 65.2 |
| 2003 | 96.1 | 68.5 |
| 2004 | 95.0 | 72.3 |
| 2005 | 89.6 | 71.9 |
| 2006 | 99.2 | 73.0 |
| 2007 | 93.7 | 80.4 |
| 2008 | 93.9 | 78.3 |
| 2009 | 92.5 | 83.2 |
| 2010 | 95.3 | 80.2 |
| 2011 | 94.9 | 80.6 |
| 2012 | 97.2 | 84.5 |
| 2013 | 100.0 | 91.4 |
| 2014 | 98.7 | 90.1 |
| 2015 | 98.3 | 92.7 |
| 2016 | 95.2 | 87.9 |
| 2017 | 96.4 | 90.3 |
| 2018 | 95.1 | 95.8 |
| Total | 94.9 | 78.5 |
|  |  |  |
|  |  |  |
|  |  |  |
| **Supplemental Table 2B: MIBB Percentage According to Urban/Rural Status of the Healthcare Facility** | | |
| Year | Urban (%) | Rural (%) |
| 1999 | 86.9 | 60.6 |
| 2000 | 93.4 | 59.9 |
| 2001 | 90.6 | 65.1 |
| 2002 | 94.0 | 64.9 |
| 2003 | 93.5 | 65.1 |
| 2004 | 92.7 | 69.6 |
| 2005 | 90.0 | 69.0 |
| 2006 | 93.2 | 71.2 |
| 2007 | 91.3 | 77.7 |
| 2008 | 91.4 | 75.3 |
| 2009 | 92.2 | 79.3 |
| 2010 | 95.2 | 75.6 |
| 2011 | 89.8 | 79.0 |
| 2012 | 95.5 | 82.1 |
| 2013 | 91.9 | 91.8 |
| 2014 | 92.2 | 89.6 |
| 2015 | 93.2 | 92.3 |
| 2016 | 92.7 | 85.1 |
| 2017 | 90.5 | 91.3 |
| 2018 | 92.5 | 94.8 |
| Total | 91.9 | 73.1 |

| **Supplemental Table 3: MIBB Percentage by Year and Facility** | | | | |
| --- | --- | --- | --- | --- |
| Facility | 1999-2003 | 2004-2008 | 2009-2003 | 2014-2018 |
| 1 | 91.6% | 91.8% | 93.0% | 92.3% |
| 2 | 71.5% | 74.2% | 64.9% | 79.2% |
| 3 | 43.8% | 51.1% | 79.6% | 100.0% |
| 4 | 75.5% | 89.4% | 90.5% | 97.4% |
| 5 | 45.2% | 52.9% | 96.3% | 95.8% |
| 6 | 58.7% | 68.9% | 78.1% | 70.0% |
| 7 | 4.8% | 8.6% | 24.2% | 70.6% |
| 8 | 78.0% | 89.0% | 88.2% | 89.7% |
| 9 | 78.0% | 80.2% | 91.5% | 93.6% |
| 10 | 47.8% | 64.0% | 96.8% | 100.0% |
| 11 | 69.8% | 74.3% | 67.1% | 86.8% |
| 12 | 10.5% | 52.0% | 83.3% | 83.3% |
| 13 | 11.8% | 92.3% | 76.7% | 95.2% |
